# Endoscopic Full Thickness Resection Device (FTRD^®^) for the Management of Gastrointestinal Lesions: Current Evidence and Future Perspectives

**DOI:** 10.3390/diagnostics15070932

**Published:** 2025-04-04

**Authors:** Magdalini Manti, Apostolis Papaefthymiou, Spyridon Dritsas, Nikolaos Kamperidis, Ioannis S. Papanikolaou, Konstantina Paraskeva, Antonio Facciorusso, Konstantinos Triantafyllou, Vasilios Papadopoulos, Georgios Tziatzios, Paraskevas Gkolfakis

**Affiliations:** 1 Gastroenterology Unit, St Mark’s Hospital, Acton Ln, London NW10 7NS, UKnkamperidis@yahoo.com (N.K.); 2 Department of Gastroenterology, General University Hospital of Larissa, 41334 Larissa, Greece; appapaef@hotmail.com (A.P.); vaspapadopoulos82@gmail.com (V.P.); 3 Transplant Unit, 1st Surgical Department, Evangelismos General Hospital, 10676 Athens, Greece; spdritsas@yahoo.com; 4 Hepatogastroenterology Unit, Second Department of Internal Medicine-Propaedeutic, Attikon University Hospital, Rimini 1, Chaidari, 12462 Athens, Greece; ispapn@hotmail.com (I.S.P.); ktriant@med.uoa.gr (K.T.); 5 Department of Gastroenterology, “Konstantopoulio-Patision” General Hospital of Nea Ionia, 14233 Athens, Greece; konparaskeva@gmail.com (K.P.); g_tziatzios@yahoo.gr (G.T.); 6 Gastroenterology Unit, Department of Surgical and Medical Sciences, University of Foggia, 71122 Foggia, Italy; antonio.facciorusso@virgilio.it

**Keywords:** full thickness resection device, FTRD^®^, EFTR, gastrointestinal lesions, endoscopic resection

## Abstract

Endoscopic full-thickness resection (EFTR) has emerged as a transformative technique for managing gastrointestinal (GI) lesions, previously deemed unsuitable for endoscopic removal. Unlike conventional endoscopic resection methods, such as endoscopic mucosal resection (EMR) and endoscopic submucosal dissection (ESD), EFTR enables en bloc excision of both intraluminal and subepithelial lesions by resecting all layers of the GI wall, followed by defect closure to prevent complications. The introduction of the full-thickness resection device (FTRD^®^) has significantly enhanced the feasibility and safety of EFTR, particularly in the colon and upper GI tract, with increasing adoption worldwide. This review provides a comprehensive analysis of FTRD^®^, focusing on its clinical applications, procedural methodology, and comparative efficacy against other endoscopic resection techniques. The indications and contraindications for EFTR are explored, highlighting its utility in treating non-lifting adenomas, subepithelial tumours, and T1 carcinomas without lymph node involvement. This review synthesizes current clinical data and FTRD^®^ advantages. Despite its strengths, EFTR via FTRD^®^ incorporates challenges such as limitations in lesion size, procedural complexity, and potential adverse events. Strategies for overcoming these challenges, including hybrid techniques and modifications in procedural approach, are examined. The review also emphasizes the need for further research to optimize surveillance strategies and determine the long-term clinical impact of EFTR in GI lesion management. By integrating recent evidence, this paper provides valuable insights into the evolving role of EFTR in therapeutic endoscopy.

## 1. Introduction

Therapeutic endoscopic techniques have significantly evolved in recent years. In addition to endoscopic mucosal resection (EMR) and endoscopic submucosal dissection (ESD), endoscopic full-thickness resection (EFTR) has emerged as a relatively new method for the removal of intraluminal and subepithelial lesions [1]. EFTR involves the segmental resection of the mucosa, submucosa, muscularis propria, and, where present, the serosa. The procedure targets the lesion along with a small margin of surrounding tissue to ensure clear resection margins. Following tissue removal, the remaining gastrointestinal wall is retracted and closed using clips to prevent peritonitis. EFTR was first introduced by Suzuki [2] in 1998 for the treatment of early gastrointestinal tumours using a ligation device, and was subsequently demonstrated in experimental models in 2001 [3,4]. At that time, lesions smaller than 10 mm, located in the rectum and duodenum, were excised, but laparoscopic intervention was required to suture micro-perforations.

Today, two types of endoscopic full-thickness resection (EFTR) are available: exposed and non-exposed. The exposed technique carries a significantly higher risk of intra-abdominal leakage, making non-exposed EFTR the safer alternative [5]. Non-exposed EFTR is performed using an over-the-scope clip (OTSC; Ovesco Endoscopy GmbH, Tübingen, Germany) or the OverStitch System (Apollo Endosurgery, Austin, TX, USA), which utilizes surgical threads. In recent years, a dedicated full-thickness resection device (FTRD^®^; Ovesco Endoscopy GmbH, Tübingen, Germany) has become available, further facilitating EFTR via OTSC [6]. However, the FTRD^®^ only entered the European market in September 2014 and was introduced in the United States for colorectal EFTR in 2017 [7,8]. It received U.S. Food and Drug Administration approval for use in the upper gastrointestinal (GI) tract in 2020 [9].

EFTR offers an effective treatment option for lesions that were previously considered unsuitable for resection with traditional methods due to their depth of invasion, or with ESD due to the prolonged procedure time. The direct tissue apposition achieved with the FTRD^®^ device minimizes the risk of exposing the peritoneal cavity to luminal contents, thereby reducing the incidence of perforation and peritonitis compared to ESD [10]. This technique also promotes faster recovery, which is particularly beneficial for elderly or frail patients. In comparison to ESD, EFTR involves a shorter learning curve [5], making it a more accessible option for endoscopists.

This review aims to provide a comprehensive overview of FTRD^®^, focusing on its clinical applications, technical aspects, and advantages over traditional resection techniques. It explores the indications and contraindications, highlighting scenarios where FTRD is most effective and cases where alternative approaches may be more suitable. Additionally, the review examines the procedural methodology, detailing the technical requirements and operational considerations necessary for successful implementation. The efficacy of FTRD is assessed through an analysis of clinical outcomes, comparing its performance with previous techniques. Furthermore, this study discusses reported adverse events and procedural challenges, offering insights into the safety profile and limitations of FTRD. By synthesizing current evidence, this review aims to provide clinicians with a deeper understanding of FTRD’s role in modern gastroenterology and its potential impact on patient management.

## 2. Technical Description of FTRD^®^

**Figure 1:**
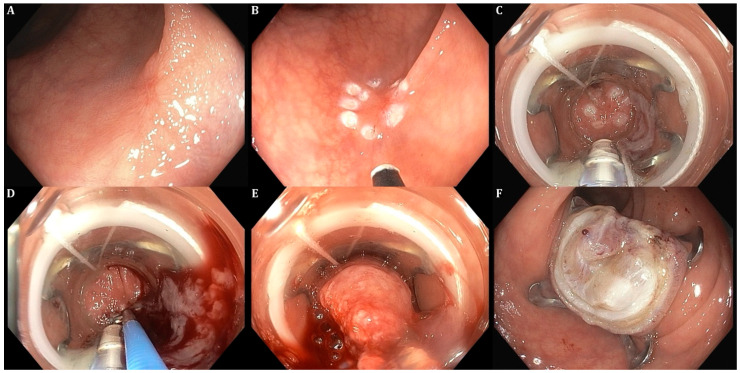
(**A**) Visualisation of a recurrent adenoma in sigmoid colon; (**B**) marking of the lesion using argon-plasma coagulation; (**C**–**E**) grasping and mobilizing the lesion within the device using suction and forceps—ensuring the lesion is completely in the cap and applying the clip, using the dedicated wheel attached to the scope; (**F**) resecting the tissue above the clip arms and retrieving a specimen (all photos are courtesy of the authors).

The FTRD^®^ technique involves full-thickness resection, resulting in serosa-to-serosa apposition. Consequently, this process creates intestinal wall duplication, effectively isolating the target lesion and minimizing the risk of perforation (Figure 1).

### 2.1. Device Description

The colonic FTRD^®^ is compatible with endoscopes that have a diameter between 11.5 mm and 13.2 mm and a working channel of at least 3.2 mm [11]. The system includes a 14 mm nickel–titanium alloy (Nitinol) over-the-scope clip (OTSC) equipped with a transparent cap and a 14 mm monofilament snare integrated at the cap’s tip [12]. The cap has a diameter of 21 mm and a depth of 23 mm. Additionally, the system features an endoscope sleeve and adhesive tapes that serve a dual purpose: securing the snare in place and preventing snare twisting or tissue entrapment. Unlike conventional snares, this snare is not advanced through the scope’s working channel but remains on the outer surface of the scope, housed within the plastic endoscope sleeve (sheath) [8].

In the upper GI tract, a dedicated gastroduodenal FTRD^®^ system has been developed for use with gastroscopes [12]. This system is smaller in size and incorporates a 20 mm balloon and a guidewire to facilitate passage through the gastroesophageal junction and pylorus. The cap has a diameter of 19.5 mm and a length of 23 mm. Jariwala et al. [13] described the use of an inflated balloon to assist in advancing the FTRD^®^ past the cricopharyngeus. This method involved balloon deflation and removal from the channel upon reaching the proximal oesophagus.

The gastroduodenal FTRD^®^ is compatible with endoscopes that have a diameter between 10.5 mm and 12 mm and a working channel of at least 3.7 mm [11]. Additionally, an anchor or tissue helix device is available to facilitate tissue mobilization [14].

### 2.2. FTRD^®^ Technique

The colonic procedure is typically performed following complete bowel preparation to ensure optimal visualization [5]. Once the lesion is identified, demarcation is carried out using techniques such as soft coagulation or argon plasma coagulation [8,15].

The next step involves grasping the lesion using either conventional grasping forceps or a dedicated grasping device. Minimal suction should be applied at this stage; instead, the tissue should be carefully drawn into the cap through gentle rotation until all the marking sites are visible within the cap.

To properly position the tissue within the cap prior to resection, gentle manoeuvres are required. Rather than relying on suction, the lesion should be carefully drawn into the cap. Excessive suction may compromise extracolonic structures and should therefore be minimized [15]. Once the target tissue is positioned within the FTRD^®^ cap and secured, the endoscopist deploys the OTSC by rotating the hand wheel. The lesion is then resected by closing the preloaded snare while applying EndoCut Q current, following a technique similar to conventional cap-assisted EMR. The resected tissue is retrieved within the cap, held by the grasper, and the scope is withdrawn. These steps should be performed consecutively and relatively quickly to prevent tissue detachment between clip deployment and snare closure [15]. After detaching the FTRD^®^ system, reintroducing the scope is generally recommended to inspect the resection site [15]. During this inspection, partial resection may be identified, as reported by Schmidt et al. [16]. This is primarily associated with lesion characteristics such as larger size, right-sided colonic tumours, and underlying tissue fibrosis. With respect to lesion size, EFTR alone is not suitable for lesions exceeding 3 cm. Partial resection may also result from technical errors during the procedure.

The estimated duration of the colonic procedure is approximately 50 min, with a reported range of 3 to 190 min. The median time required for FTRD^®^ insertion is 10 min (range 1–50), while the median resection time is 5 min (range 1–90) [16]. The FTRD^®^ procedure is exclusively performed using CO₂ insufflation, and gas leakage is recognized as a potential device-associated complication.

Advancing the endoscope can be challenging in the lower GI. During EFTR, the endoscope must be advanced three times in total, which can be particularly difficult when the FTRD^®^ system is mounted on the scope during a colonoscopy [15]. To facilitate colonic intubation, Shigeta et al. [17] suggested performing the procedure underwater. Right colonic lesions may be particularly challenging to approach and grasp due to scarring. In such cases, the prOVE cap (Ovesco Endoscopy) [8] can be used as an alternative. This cap has similar dimensions to the standard FTRD^®^ cap but lacks the mounted clip and snare. The use of the prOVE cap facilitates the assessment of the lesion’s accessibility and whether it can be successfully drawn into the cap. If feasible, the endoscope is withdrawn, and the FTRD^®^ system is then mounted and reintroduced [12]. To enhance scope advancement, inserting a guidewire into the colon before the initial scope withdrawal has been suggested. This allows the FTRD^®^ system to be advanced along the guidewire, improving lesion localization [12]. In cases where scope advancement remains difficult, switching to the gastroduodenal FTRD^®^ system has also been proposed. This device has a slightly smaller diameter (19.5 mm) compared to the colonic FTRD^®^ (21 mm), potentially easing navigation [14]. Of note, while balloon dilators are primarily used in gastroduodenal EFTR, they have also been successfully employed in cases of anal stenosis [14].

A comparable technique is utilized for upper GI lesions with the gastroduodenal FTRD^®^ system.

## 3. Indications and Contraindications of EFTR via FTRD^®^

### 3.1. Indications of FTRD^®^

EFTR can be used for upper and lower GI lesions. Regardless of the EFTR technique employed, its indications remain similar and are guided by factors related to the lesion’s characteristics or the patient’s clinical profile (Table 1).

#### 3.1.1. Lesion Characteristics

The characteristics of the lesion are key factors in determining the indication for EFTR [8]. Lesion size, in particular, is a major determinant of the appropriate endoscopic resection method. EFTR using the FTRD^®^ system has been successfully performed for lesions up to 25 mm in size [8], although Mori et al. [18] reported a mean lesion size of 20.71 mm among those eligible for EFTR. While larger polyp resections have been documented in the literature, such procedures are limited by the 21 mm cap size [19].

Although EFTR is not yet included in the European Society of Gastrointestinal Endoscopy (ESGE) guidelines, it is recommended by the American Society of Gastrointestinal Endoscopy (ASGE) guidelines [5]. EFTR is commonly performed in the stomach and colon, but it can also be safely applied to duodenal lesions. In 2022, Wang et al. [20] reported the successful application of EFTR in 14 patients with lesions at the oesophagogastric junction (GOJ). Its use, however, remains limited for lesions located in the jejunum, ileum, or in close proximity to the anal canal [5]. In a case series published by Li et al. [21], exposed EFTR was performed using balloon-assisted enteroscopy (either single or double) for small bowel lesions, with a technical success rate of 60% (3 out of 5 patients). For duodenal lesions, Bauder et al. [22] recommended that the target lesion should be at least 20 mm away from the papilla to minimize complications. EFTR is also utilized for lesions in the upper and middle rectum, as well as in the lower rectum involving the distal 5 mm [23]. Lesions in challenging anatomical sites, such as the appendiceal orifice or those located near diverticular openings, carry higher risk of adverse events, including acute appendicitis and diverticulitis [24]. Consequently, EFTR is recommended as the preferred approach in these high-risk cases.

Invasion depth is another important factor to consider when assessing the suitability of EFTR. This technique is most commonly performed for non-lifting adenomas located in fibrotic areas, whether primary or recurrent [15,19]. When deeper invasion is suspected, EFTR may be selected as a first-line resection technique. Suspicious serrated luminal lesions with associated fibrosis, which are more likely to extend into deeper layers, are also considered suitable candidates for EFTR [5]. Additionally, EFTR is indicated for subepithelial lesions such as gastrointestinal stromal tumours (GIST), neuroendocrine neoplasms (NEN), or granular cell tumours [5]. These types of lesions typically do not require lymph node removal, while the risk of tumour cell dissemination is minimal. Regarding GISTs, the ESGE guidelines recommend that lesions smaller than 2 cm can either be monitored endoscopically or removed [25]. Interestingly, Canadian consensus guidelines suggest that GISTs smaller than 1 cm should be surgically excised due to their higher metastatic risk. However, active surveillance may be considered for gastric GISTs measuring less than 2 cm [26].

T1 carcinomas without evidence of lymphatic involvement can also be safely resected using EFTR. According to the ESGE [27] and ASGE [5] guidelines, as well as the US Multi-Society Task Force recommendations [28], tumours with low-risk features—defined by the absence of lymphatic or vascular involvement, submucosal invasion of less than 1000 μm, and well-differentiated histology (grade 1 tumours)—can be safely excised endoscopically. The risk of lymph node metastasis in such cases is low (approximately 1%), and the 5-year recurrence-free survival rate exceeds 90%. Additionally, lesion location is a crucial factor in determining the most appropriate resection technique [15].

#### 3.1.2. Patient Characteristics

Patients’ frailty is crucial in determining lesion management. Hence, patients with comorbidities who do not meet the criteria for surgical intervention are candidates for endoscopic resection [5]. Patient’s preferences should also be taken into consideration.

### 3.2. Contra-Indications of FTRD^®^

Despite its merits, the use of EFTR is governed by established principles of endoscopic resection, according to the therapeutic potential and the risk of recurrence. For example, the risks outweigh the benefits in cases of metastatic cancer with required extended surgical resection along with systemic medical therapy (chemotherapy or immunotherapy). Proper endoscopist training is also needed prior to performing EFTR. EFTR contra-indications are summarised in Table 2.

## 4. FTRD^®^ Performance and Safety in Upper and Lower GI

Three key factors must be considered when evaluating FTRD^®^ efficacy. First, the technical success rate, defined as en bloc resection and macroscopically complete resection. Second, the achievement of clear histologic margin (R0 resection) [15]. Third, the accurate evaluation of the intact full-thickness sample by the pathologists. Further information regarding the identification of the studies is found in Supplementary Material S1 (Figure S1).

### 4.1. Upper GI

The FTRD^®^ has been successfully employed in treating upper GI lesions, primarily involving the stomach and duodenum. The introduction of the gastroduodenal device represented a breakthrough in managing these lesions due to its smaller diameter, making it more suitable for the narrow lumen of the duodenum compared to the larger colonic FTRD^®^.

In 2020, Meier et al. [29] published the RESET trial, a prospective study introducing a new gastroduodenal FTRD^®^. Among 29 patients with gastric subepithelial tumours, en bloc resection was achieved in 89% and R0 in 76%. Jariwala et al. [13] concluded in their study that FTRD^®^ was technically more difficult in the antrum than in the stomach body due to its wall thickness. This evidence was also supported by subsequent studies, where successful resection was achieved in 91% and R0 margins in 90%. In a retrospective study, Hajifathalian et al. [30] reported less favourable outcomes with complete resection yielded in 77% of cases and R0 in 68%. The existing data were summarized in a recent meta-analysis by Bomman et al. [31], who included 139 patients who had undergone FTRD^®^ in upper GI with en bloc resection rates of 88.2% and R0 resection recorded in 70.7%.

Potential drawbacks included the need for complementary ESD or incomplete resections during the initial procedure. Moreover, the difficulty in suctioning the lesion in the cap can make it hard to position or secure the lesion for resection, thereby necessitating an alternative approach.

### 4.2. Lower GI

FTRD^®^ has emerged as a promising tool for endoscopic full-thickness resection in the lower gastrointestinal tract, offering a minimally invasive alternative to surgery for difficult-to-resect lesions. Multiple studies have evaluated its efficacy and safety, demonstrating high rates of technical success and complete resection.

In 2018, Vitali et al. [32] conducted a prospective study involving 13 patients undergoing FTRD^®^. Concurrently, Schmidt et al. [16] published results from a German multicentre prospective trial, “WALL-RESECT,” which included 181 patients and reported an en bloc EFTR resection rate of 89.5% and an R0 resection rate of 76.9%. Notably, the R0 resection rate was significantly higher for lesions smaller than 2 cm compared to larger lesions (81.2% vs. 58.1%, *p* = 0.0038). In the United States, a multicentre cohort study [24] involving 95 patients reported an R0 resection rate of 82.7% [23]. Similarly, a recent Italian study [33] assessed 35 patients with colonic FTRD^®^, with 18 lesions located in the left colon. Both en bloc and R0 resection were achieved in 94% of cases. The study also evaluated recurrence rates at a median follow-up of three months, reporting a recurrence rate of 19.4%. Among these cases, five patients required multiple FTRD^®^ sessions, with R0 resection ultimately achieved in three of them. A Dutch study by Bronswaer et al. [34] focused exclusively on seven patients with appendiceal lesions, demonstrating a 100% technical success rate and an R0 resection rate of 85.7%. In terms of safety, two adverse events were reported: one patient with a history of appendectomy developed an abscess, which was managed conservatively, while another patient experienced appendicitis. Overall, these studies highlight the effectiveness of FTRD^®^ in achieving high rates of complete resection, particularly in smaller lesions, while also emphasizing the importance of careful patient selection to minimize complications.

Several retrospective studies have evaluated the use of FTRD^®^ in the resection of colorectal neoplasms [35]. In 2019, Velegraki et al. [36] published a retrospective study assessing 17 colorectal lesions treated with FTRD^®^, reporting an en bloc resection rate of 82.3% with R0 margins. Among carcinoma cases, R0 resection was achieved in 66.6%, while subepithelial tumors had a higher R0 rate of 83.3%. Notably, technical success was significantly greater for lesions smaller than 20 mm compared to larger lesions (*p* = 0.0429).

In a larger international retrospective study, Ichkhanian et al. [37] analysed 66 patients with lesions involving the appendiceal orifice, demonstrating an R0 resection rate of 93% and an en bloc resection rate of 80%. Similarly, a UK registry study [38] included 68 patients with various lesion types, including 29 cases of non-lifting polyps, 13 T1 tumours, 9 subepithelial lesions, and 17 appendiceal or diverticulum-associated tumours. Complete macroscopic resection was achieved in 60 cases, with an overall R0 resection rate of 76.8%. In Germany, Albrecht et al. [39] conducted a study involving 70 patients, where EFTR was successfully performed in 65 cases. Three patients with larger lesions were deemed unsuitable for FTRD^®^ resection. Among the 65 successfully resected lesions, R0 resection was achieved in 59 cases (97%). Their analysis further confirmed that histologically complete resection rates were higher for lesions smaller than 20 mm compared to those larger than 20 mm (92.9% vs. 86.5%).

Meier et al. [40] published the German colonic FTRD^®^ study in 2020, reporting a technical success rate of 88.2% and an R0 resection rate of 80% across 1178 procedures. These results align with another German retrospective case series [41] of 229 patients, which demonstrated a slightly lower technical success rate of 83.8% and an R0 resection rate of 77.2%. The minor discrepancies between these studies may be attributed to differences in patient selection, lesion characteristics, and operator experience.

In Italy, Andrisani et al. [42] conducted a multicentre study among 114 patients, achieving a notably high technical success rate of 94.4% and an R0 resection rate of 92%. These outcomes were among the highest reported in the literature, suggesting that standardized protocols and experienced centres may contribute to improved results. However, follow-up at three months revealed residual tissue in seven patients, emphasizing the need for careful post-procedural surveillance, even in cases with initially successful resections. Zwager et al. [43] studied 330 FTRD^®^ cases in 2022, reporting a technical success rate of 87% and an R0 resection rate of 85.6%. While these figures are comparable to previous studies, a notable 15.3% of patients required oncologic surgery after FTRD^®^. This highlights an important limitation of EFTR—while it achieves high success rates, a subset of patients may still require additional surgical intervention, particularly in cases where histological assessment reveals high-risk features. Focusing specifically on early colorectal cancer, Kuellmer et al. [44] analysed 156 cases treated with colonic FTRD^®^, finding a technical success rate of 92.3% and an R0 resection rate of 71.8%. Interestingly, a subgroup analysis showed that R0 resection rates were significantly higher among patients with prior incomplete polypectomy compared to those with non-lifting colonic lesions (87.5% vs. 60.9%, *p* < 0.001). This suggests that FTRD^®^ may be particularly advantageous in cases where initial resection attempts have left residual tissue, while its efficacy in truly non-lifting lesions remains more limited.

Regarding rectal neuroendocrine neoplasms (NENs), Meier et al. [45] studied 40 cases with a median lesion size of 8 mm, reporting a macroscopic resection rate of 95%. These findings reinforce FTRD^®^ as a promising option for small rectal NENs, which often require complete and precise resection due to their malignant potential. Compared to colorectal neoplasms, NENs appear to have a higher likelihood of successful removal with FTRD^®^, possibly due to their well-defined margins.

Hybrid EFTR techniques have been explored in various settings to enhance the resection of complex lesions. Early studies combined EFTR with EMR for the treatment of rectal recurrent adenomas with surrounding fibrosis [46], demonstrating its potential for lesions with challenging morphology. The first reported hybrid EFTR-ESD procedure was described by Lupu et al. [47] in 2018 for an appendiceal adenoma. This was followed by a case study from Andrisani et al. [48] in 2020, further highlighting the feasibility of combining EFTR with advanced resection techniques. Previously, Andrisani et al. [49] had also explored EFTR in combination with ESD, suggesting that hybrid approaches could enhance complete resection rates for select lesions.

A more recent retrospective study [50] systematically evaluated hybrid EFTR, integrating it with either EMR or ESD for lesions exceeding 20 mm in size (41.7 mm in the EFTR+EMR group and 31.7 mm in the EFTR+ESD group). The strategy involved initiating resection with EMR or ESD, followed by FTRD^®^ application to reduce lesion size to 25 mm, making complete resection more manageable. The procedural steps outlined by Tribonias et al. [50] involved submucosal injection with a solution containing saline, epinephrine, and indigo carmine for EMR, followed by resection using a 15 mm or 25 mm polypectomy snare. In hybrid procedures with ESD, a needle-type endoscopic knife was utilized. Once the lesion’s diameter was reduced to 20 mm, FTRD^®^ was applied. This process required withdrawal of the endoscope, attachment of the FTRD^®^, and subsequent reinsertion of the scope, as previously described. The hybrid technique was successfully completed in 14 out of 16 patients, though in two cases, scope advancement with the FTRD^®^ was not feasible due to a fixed sigmoid, illustrating potential anatomical limitations of the approach.

In 2023, a German retrospective study [51] evaluated hybrid EFTR-EMR in 75 patients, reporting an en bloc resection rate of 97.3%. This study served as a follow-up to an earlier retrospective analysis from the same group published in 2017 [52], further solidifying the role of hybrid techniques in endoscopic full-thickness resection. Meier et al. [51] employed similar procedural steps for hybrid EMR and FTRD as suggested by Tribonias et al. [50]; however, their injection solution contained methylene blue rather than indigo carmine. Crucially, the resection must be initiated from the lesion’s periphery to ensure that the central non-lifting area—in the case of laterally spreading tumours—or the non-lifting tumour base—in sessile polyps—remains accessible for successful FTRD^®^ application. Similarly, Bauermeister et al. [53] investigated EFTR-EMR in a smaller cohort of 17 patients in 2021, while during the same year, American gastroenterologists applied the hybrid technique [54] in a larger group of 69 patients. The consistency in high technical success across multiple studies suggests that hybrid EFTR approaches may be particularly beneficial for lesions that exceed standard FTRD^®^ size limitations.

Several meta-analyses and systematic reviews have evaluated the efficacy and safety of FTRD^®^ in colorectal lesion management. In 2021, a meta-analysis by Li et al. [55] analysed nine studies comprising 469 patients, reporting a high technical success rate of 94% and an R0 resection rate of 84.9%, reinforcing the effectiveness of FTRD^®^ in achieving complete resections. That same year, Fahmawi et al. [56] conducted a systematic review of 555 lesions treated with FTRD^®^, finding comparable en bloc (89.25%) and R0 resection rates (82.4%). Notably, for peri-appendicular lesions (*n* = 44), acute appendicitis occurred in 19.7%, indicating a specific risk associated with this anatomical location.

Larger-scale analyses have further validated these findings. Dolan et al. [57] studied 1936 patients, of whom 1889 underwent EFTR via FTRD^®^, demonstrating technical success in 87.2% and margin-negative (R0) resection in 78.4%. Similarly, a meta-analysis focusing specifically on colonic FTRD^®^ [58] included 1538 cases, with reported technical success of 90% and an R0 resection rate of 77.8%. These findings align with previous studies, suggesting that while EFTR with FTRD^®^ is highly effective, achieving clear margins in larger or complex lesions remains challenging.

In 2020, Brewer et al. [19] conducted a meta-analysis encompassing 18 studies with a total of 730 patients presenting with both upper and lower gastrointestinal lesions. The analysis reported an en bloc resection rate of 95% (95% CI: 92–96) and an R0 resection rate of 82% (95% CI: 75–89), reinforcing the efficacy of EFTR in achieving complete resection across a diverse range of lesions.

Hybrid techniques have also been systematically assessed. Fakhoury et al. [59] performed a meta-analysis of hybrid EFTR-EMR involving 244 patients, reporting an impressive success rate of 97% and an R0 resection rate of 88%, indicating that hybrid approaches may offer an advantage in certain cases.

Comparative studies between FTRD^®^ and other resection methods provide additional insights. A retrospective study by Yzet et al. [60] compared FTRD^®^ to ESD in 275 patients with residual colorectal cancer post-EMR. Among them, 177 underwent ESD and 98 underwent FTRD^®^, with similar R0 resection rates (83.3% vs. 77.6%, *p* = 0.25). However, lesions treated with ESD were significantly larger (*p* < 0.001), and the adverse event rate was higher in the ESD group (16.3% vs. 5.1%), suggesting a potential safety advantage of FTRD^®^ despite its lower utilization for larger lesions. However, the study’s direct comparison was limited due to inclusion of unambiguous lesions.

A more recent meta-analysis by Singh et al. [61] compared EFTR via FTRD^®^ (215 patients) and ESD (315 patients), finding similar en bloc (*p* = 0.31) and R0 resection rates (*p* = 0.42). However, EFTR demonstrated significantly shorter procedure times (*p* = 0.004) and fewer complications (*p* < 0.00001), reinforcing its role as a safer and more efficient alternative in select cases.

Andrisani et al. [62] conducted a multicentre randomized trial involving 90 patients with challenging colorectal lesions, defined as adenomatous recurrences, non-lifting adenomas, and nongranular laterally spreading tumours. The study compared FTRD^®^ and ESD for lesions smaller than 30 mm, as both procedures were considered suitable for these cases. The researchers found en bloc resection rates of 95.5% for FTRD^®^ and 93.3% for ESD. R0 resection rates were comparable between the two groups—FTRD^®^ vs. ESD: 42 (93.3%) vs. 36 (80%); *p* = 0.06. Adverse events and procedure times were lower in the FTRD^®^ group, although these differences did not reach statistical significance (4.44% vs. 15.5%, *p* = 0.04 and 25.6 ± 10.6 min vs. 76.7 ± 26.4 min, *p* ≤ 0.01). A summary of the comparative studies is included in Table 3.

FTRD^®^ has been compared to transanal endoscopic microsurgery (TEM) for the treatment of rectal NETs in a German retrospective study by Brand et al. [63]. The study included a similar number of patients in each group (15 in the EFTR group and 13 in the TEM group), and results demonstrated no significant differences in en bloc resection rates or R0 resection rates, suggesting that both techniques are equally effective in achieving complete tumour removal. A broader comparative analysis by Bisogni et al. [64] evaluated FTRD^®^, TEM, and ESD for rectal lesion resection in 76 cases. While en bloc resection rates were higher in the FTRD^®^ and TEM groups compared to ESD, R0 resection percentages remained similar across all techniques. Notably, perforation rates were lower in the FTRD^®^ and TEM groups, indicating a potential safety advantage over ESD. However, no significant differences were observed in bleeding or post-polypectomy syndrome, highlighting that all three techniques carry comparable risks regarding post-procedural complications. These findings reinforce FTRD’s ^®^ position as a viable and potentially safer alternative for rectal NET resection, particularly in cases where minimizing perforation risk is a priority.

The collective findings from retrospective studies, multicentre trials, and meta-analyses highlight the high technical success and R0 resection rates associated with FTRD^®^, particularly for colorectal neoplasms and rectal NETs. Comparative studies suggest that while FTRD^®^ performs similarly to ESD and TEM in terms of en bloc and R0 resection rates, it may offer advantages in terms of procedure time and safety.

## 5. Adverse Events and Technical Failure

FTRD^®^ is a valuable tool in advanced endoscopic resection; however, its use can be associated with complications. In the study of Ichkhanian et al. [24] which included 95 patients, adverse events were found in 5.3% of the patients during EFTR, while 2.1% required surgical intervention. Complications may include anal trauma after cap insertion, bleeding, luminal oedema, post-polypectomy syndrome (entailing symptoms of fever, abdominal pain, increase of white blood count), perforation, and acute appendicitis [8,14]. Bleeding rates have been calculated to be around 2.2% in a meta-analysis [55], while delayed post-polypectomy syndrome has been reported [15].

Appendicitis rates post-FTRD^®^ are up to 17% [37]. Prophylactic antibiotics periprocedural do not eliminate the risk and are not suggested [65]. When EFTR was performed in 66 patients with lesions involving the appendiceal orifice, 17% developed appendicitis, while 10% (6 patients) required surgical appendectomy [37]. In 2023 a meta-analysis for lesions involving the appendiceal orifice [66] studied the post-resection appendicitis rates. It was found that 15% of patients developed appendicitis (29 out of 205) with 61% of which requiring surgery.

The immediate clip deployment of FTRD^®^ allows for lower perforation rates, in comparison to ESD [15]. In the study of Albrecht et al. [39], perforation of the sigmoid was found in 1 out of 65 patients. However, delayed perforation cases have been reported even 7 days post-procedure [67]. Post-EFTR surgery was also studied in the meta-analysis by Brewer et al. [19] with <0.1% pooled rates in cases of both upper and lower EFTR. Formation of entero-colonic fistula has also been described in the literature, post-FTRD^®^ due to entrapment of small bowel during clip deployment [16,68]. Oesophageal tears post-FTRD^®^ could occur when treating upper GI lesions and these were reported in 2 out of 22 patients in a retrospective study [69]. Of note in this study, the balloon was not used during FTRD^®^ insertion, which could potentially hinder these events.

In a German FTRD^®^ registry [40], device failure has also been reported. Cases of clip or snare malfunction leading to perforation or failure of resection have been published. Andrisani et al. [42] reported device failure in 11% of patients who had FTRD^®^ due to colorectal lesions. In cases where the integrated snare fails to cut following OTSC clip deployment, the device is typically withdrawn, and resection is completed using a conventional snare [42]. However, it is important to note that employing a conventional snare in close proximity to the OTSC clip may increase the risk of thermal injury and tissue necrosis, potentially resulting in delayed perforation. This risk is minimised when utilizing the integrated FTRD snare, as its distance from the deployed clip is predetermined and optimally adjusted to ensure safer resection [70]. Cases of clip mis-deployment and incomplete OTSC closure in the transverse colon, resulting in a mucosal leak, have been reported. In these instances, the leak was successfully managed by placing a standard OTSC [42]. A comparable case [70] involved a failed OTSC deployment, which led to a perforation that was subsequently closed with a secondary OTSC. However, this delayed closure ultimately resulted in peritonitis. In a retrospective study among 17 Italian hospitals, technical failure was identified in 77 out of 750 patients. The commonest issues were the noncutting snare (53%), clip mis-deployment (31%) and cap misplacement (16%) [71]. When the latter occurs, a guidewire can be utilized in advance to facilitate the introduction of the cap to the lesion site [12].

As described previously, the FTRD^®^ system includes a grasper for lesion retraction. Due to the initial grasping of the mucosa, the tissue could be damaged and mislead the histology [18]. A case of tissue grasping failure has also been reported and the subsequent use of a snare was described in the literature [13] for GIST resection. Jariwala et al. [5] used a snare for mucosal resection and subepithelial lesion exposure. The latter was then retained in the cap using tissue helix rather than grasper. Consequently, the clip was applied, and resection was completed using electrical current. In some cases, the provided grasping forceps may fail to adequately secure the lesion and may inadvertently cause tissue tearing during retraction into the cap. When this occurs, an alternative strategy involves grasping a different portion of the lesion, ideally at the base or along the right/bottom edge, while applying steady traction, minimal suction, and gentle endoscope manoeuvring to facilitate safe and effective retraction [14]. For subepithelial lesions, the OTSC anchor device (Ovesco AG) may serve as an alternative tool, offering improved tissue manipulation and enhancing the likelihood of successful lesion capture and resection [22]. In cases of technical failure, it is crucial to ensure that any entrapped tissue is subsequently removed to prevent complications. This can be achieved using a hot snare and hot biopsy forceps employing the hot avulsion technique following the reinsertion of the colonoscope [54]. This method facilitates precise tissue removal while minimizing the risk of residual tissue or incomplete resection. Except tissue grasping, defect closure could be challenging as well during FTRD^®^ in non-linear areas.

So far, the largest registry for adverse events post FTRD^®^ was studied by Zwager et al. [70] and published in 2023. It included 1892 procedures, 213 of which had an adverse event. Forty-seven patients had perforation (2.5%) and when examining the timing of the adverse event, 27 cases were immediate (57.4%) and 20 delayed. Endoscopic closure was consecutively achieved in 29.8% of these cases, while 4.3% required only antibiotic treatment for managing perforations. Appendicitis occurred in 7 cases, delayed bleeding in 1 case, luminal stenosis in 2 cases and post-procedure pain in 1 (lesion proximal to the dentate line). Equipment dysfunction was reported in 1 case with grasper entrapment in the clip. Appendicitis occurred in 13 out of 131 cases located in the appendiceal orifice, 7 of which required surgical intervention.

## 6. Strengths and Challenges of FTRD

FTRD^®^ provides a valuable solution for lesions previously deemed unresectable due to depth of invasion or the long procedure times required for EMR and ESD. It minimizes the risk of complications such as perforation and peritonitis by directly apposing tissue during resection. Moreover, FTRD^®^ offers a quicker recovery and fewer complications, making it an appealing option, especially for elderly or frail patients who are at higher risk for surgery. Procedure times range from a minimum of 40 min to a maximum of 105 min. Given the operative time for laparoscopic partial gastrectomy, the maximum procedure time of 105 min for EFTR is considered acceptable. Various factors, including tumour characteristics and the operator’s experience, can influence the procedure duration [18].

Although FTRD^®^ is not yet formally included in current ESGE guidelines [72], its use is advocated by the authors due to the lower complication rates [62] and reduced procedure times [61]. FTRD^®^ is particularly recommended for cases where deeper tumour infiltration is suspected. Conversely, lesions in the rectum are generally advised to be resected via ESD, given the current lack of supporting evidence for FTRD^®^. Similarly, evidence regarding FTRD^®^ use in oesophageal and duodenal lesions remains inconclusive. Authors suggest that FTRD^®^ may be appropriate for colonic lesions located in accessible anatomical sites, to mitigate ESD-related complications. However, its use should be guided by the endoscopist’s expertise and the availability of equipment. Further robust evidence and formal guidelines are needed to support these suggestions.

EFTR is considered cost-effective when compared to traditional surgical treatments. This minimally invasive approach reduces healthcare costs by avoiding lengthy hospital stays and the associated complications of surgery. As reported by Gibiino et al. [71], there is currently no reimbursement available for the device in Italy. Nevertheless, Kuellmer et al. [73] conducted a cost-effectiveness analysis using data from the “WALL-RESECT” study [16], incorporating a simulated comparison arm that included standard endoscopic resection (SER) treatments such as EMR, ESD, and surgery, with R0 resection serving as the measure of effectiveness. The efficacy of these alternative modalities was estimated based on published outcomes and lesion characteristics. Their analysis revealed that while the cost per case was EUR 2852.20 for the EFTR group, EUR 1712 for the SER group, and EUR 8895 for the surgical resection group, the incremental cost-effectiveness ratio (ICER)—representing the additional cost per R0 resection compared with EFTR—was EUR 5196.47 for non-EFTR procedures and EUR 26,533.13 for surgical resection. These findings underscored the cost-effectiveness of EFTR. However, the cost-effectiveness of FTRD^®^ specifically is still being evaluated, and further research is needed to confirm its long-term financial benefits in comparison to conventional surgical methods [73].

Despite its advantages, FTRD^®^ has some limitations. First, its use is limited to lesions up to 20 mm. Larger lesions can only be managed via hybrid techniques using ESD. However, Fraile Lopez et al. [74] suggested that neoadjuvant chemotherapy could decrease the lesion size to allow for FTRD^®^ application. Still, this comes with concomitant increased risk of delayed perforation. In terms of mucosal inspection, the cap is 23 mm in length, obscuring the view during the procedure.

Because of its complexity in both equipment setup and resection technique, endoscopists must become familiar with the procedure before performing EFTR. Similar to other endoscopic devices and techniques like ESD and EMR, EFTR with the FTRD^®^ demands proper training and endoscopic expertise. Completion of required training is necessary before acquiring and using the FTRD^®^ [12]. However, the learning curve is shorter than exposed EFTR and ESD [5]. In addition to endoscopist’s training, endoscopy nurses also need to be familiar with the technique. It is crucial to highlight that clip placement should be promptly followed by snare resection, which often requires effective communication and collaboration between the endoscopist and the assistants handling the snare and grasper. The endoscopist should activate the clip, while two separate assistants should manage the grasper and snare. Any delay in snare resection after clip placement, slippage of the lesion from the grasper, or performing snare resection before firing the clip can lead to incomplete resection or immediate complications [12].

Surveillance intervals and recurrence rates remain an uncertainty when performing EFTR due to lack of sufficient data. Li et al. [55] studied the rates of residual and recurrent adenomas among 469 patients, with 8.5% of them having detectable lesions during the surveillance colonoscopy. For surveillance optimisation, the resected area should be thoroughly inspected [12]. Post-EFTR, the clip is detached in 3 months for 70%, allowing for further future surveillance. However, if remaining in situ, it is removed by a bipolar cutting device [75]. Then, in case of recurrence, a follow-up EFTR, EMR, or ESD can be attempted [11]. FTRD^®^ clips can also be removed via the remove system via clip fragmentation [76]. In upper GI lesions, ESGE guidelines [72] recommend surveillance gastroscopy at 3–6 months after ESD, followed by annual endoscopy for the first five years. Authors propose that similar surveillance intervals should be applied following FTRD^®^ procedures. For colonic lesions, surveillance endoscopy is advised at 12 months, utilizing high-definition white-light endoscopy and chromoendoscopy with biopsies taken only from suspicious areas. Subsequent surveillance should adhere to established colorectal cancer guidelines. In cases where post-FTRD histology reveals a positive lateral margin without evidence of lymphovascular invasion, a follow-up examination with high-definition chromoendoscopy (either virtual or dye-based) combined with targeted biopsies is recommended at 3–6 months. For lesions located in the duodenum or small bowel, surveillance intervals should be determined based on colonic surveillance protocols. Generally, in the absence of relevant evidence, guidelines should be followed based on the pathology and the characteristics of the lesion rather than the specific technique used.

Further research is needed regarding the best option when treating recurrence. A Danish case series [77] followed up 23 patients post EFTR in 12 months. Among 10 patients with malignant lesions, one patient had histological relapse, while none of the 12 patients with benign initial findings had residual tissue at follow-up. Gericke et al. [78]. published a case report of repeat FTRD^®^ during surveillance gastroscopy for a previous duodenal adenoma.

## 7. Conclusions

EFTR facilitated by FTRD^®^ has significantly expanded the therapeutic options for GI lesions. This approach offers a minimally invasive alternative to surgery, particularly for frail patients or those with non-lifting adenomas, subepithelial tumours, and early-stage carcinomas. The technique demonstrates high rates of en bloc and R0 resection, especially for lesions under 20 mm, though its efficacy for larger lesions is improved through hybrid methods. While EFTR reduces complications, such as perforation, compared to ESD, challenges remain, including device failure, lesion size limitations, and the potential for appendicitis or fistula formation. Proper endoscopist training, patient selection, and procedural expertise are critical for success. Further studies are needed to address surveillance intervals, recurrence rates, and innovations to mitigate post-procedural complications. Overall, EFTR represents an efficient approach, with promising applications for both therapeutic and diagnostic GI interventions.

## Figures and Tables

**Table 1:** EFTR indications associated with the lesion and patient characteristics.

Lesion Characteristics	Patient Characteristics
1. Size up to 20 mm	A. High frailty score
2. Lesions located in stomach, duodenum, or colon	B. Personal preferences
3. Non-lifting lesions (primary or recurrence)	
4. SEL (subepithelial lesions): e.g., GIST, NET, granular cell tumours	
5. Early T1 carcinomas	
6. Adenomas at difficult anatomic sites (appendiceal orifice, diverticulum)	

**Table 2:** EFTR contra-indications associated with the lesion, patient and endoscopist characteristics.

Lesion Characteristics	Patient Characteristics	Endoscopist Characteristics
Lesion less than 20 mm from the papilla	Inadequate bowel preparation	Inadequate training
Metastatic lesion	Inadequate sedation	Faulty equipment

**Table 3:** Comparative studies between FTRD and ESD in terms of en bloc resection, R0 resection and adverse events.

Authors	Endoscopic Modality	Number of Patients	En Bloc Resection	Ro Resection	Adverse Events
Yzet et al. [60]	FTRD	177	93.8%	83.3%	5.1%
	ESD	98	98.3%	77.6%	16.3%
Singh et al. [61]	FTRD	215	94%	84%	6.5%
	ESD	315	91%	80%	21.3%
Andrisani et al. [62]	FTRD	45	95.5%	93.3%	4.44%
	ESD	45	93.3%	80%	15.5%

## Supplementary Materials

The following supporting information can be downloaded at: https://www.mdpi.com/article/10.3390/diagnostics15070932/s1, Figure S1: PRISMA flow diagram illustrating methodology of literature search.
