# Supplementary figures and images for: Endoscopic Full Thickness Resection Device (FTRD®) for the Management of Gastrointestinal Lesions: Current Evidence and Future Perspectives
Source: Diagnostics (Basel). 2025 Apr 4;15(7):932. doi: 10.3390/diagnostics15070932 (PMC11988833; doi:10.3390/diagnostics15070932)

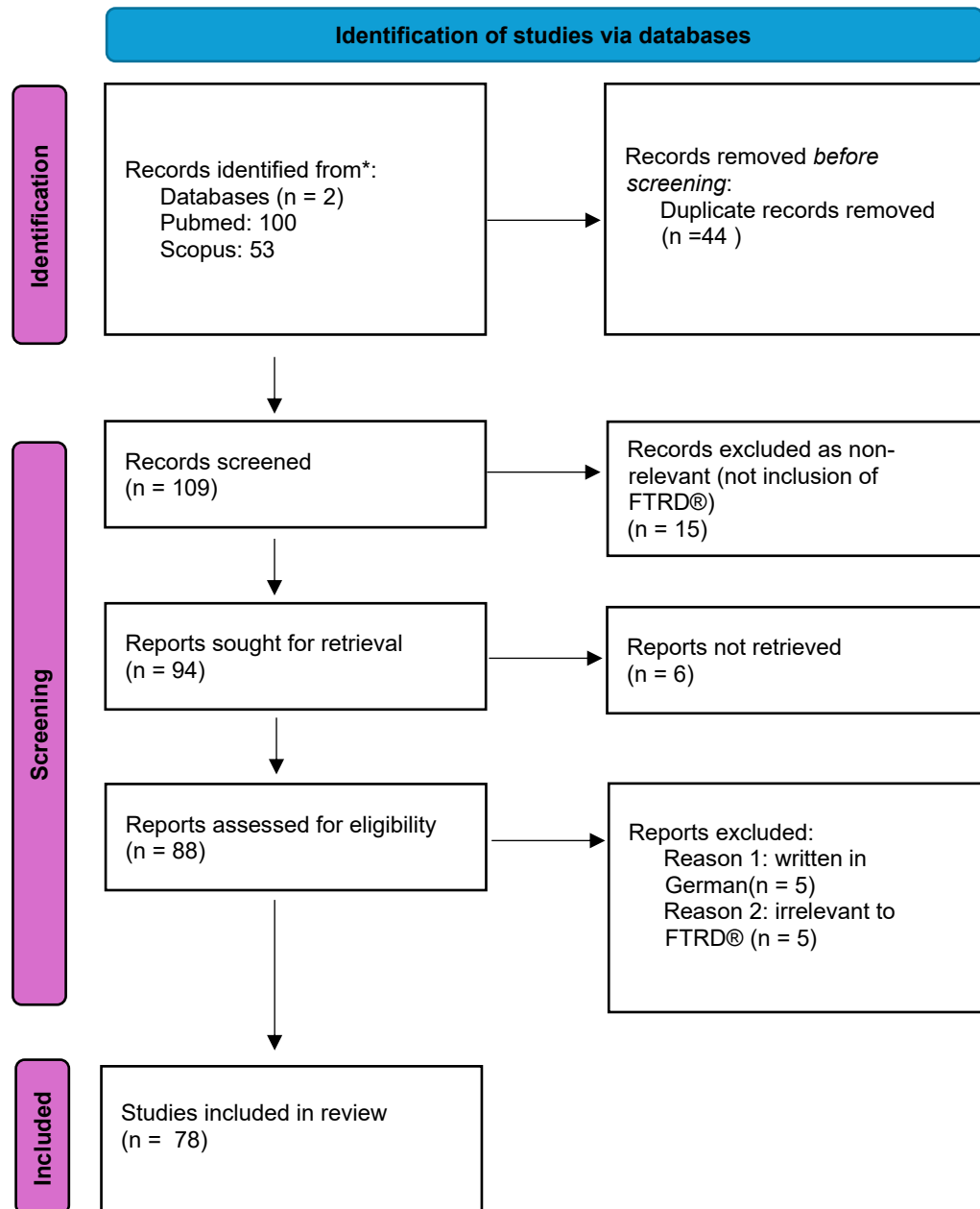

**Figure S1.** PRISMA flow diagram illustrating methodology of literature search.

Supplement: Supplementary file 1 [file diagnostics-15-00932-s001.zip › diagnostics-3527242-supplementary.pdf]
